# Anlotinib has good efficacy and low toxicity: a phase II study of anlotinib in pre-treated HER-2 negative metastatic breast cancer

**DOI:** 10.20892/j.issn.2095-3941.2020.0463

**Published:** 2021-08-15

**Authors:** Nanlin Hu, Yiran Si, Jian Yue, Tingting Sun, Xue Wang, Zhuqing Jia, Songlin Gao, Qiao Li, Yang Shao, Jiayu Wang, Yang Luo, Fei Ma, Binghe Xu, Peng Yuan

**Affiliations:** 1 Department of Medical Oncology, National Cancer Center/National Clinical Research Center for Cancer/Cancer Hospital, Chinese Academy of Medical Sciences and Peking Union Medical College, Beijing 100021, China; 2 Department of VIP Medical Services, National Cancer Center/National Clinical Research Center for Cancer/Cancer Hospital, Chinese Academy of Medical Sciences and Peking Union Medical College, Beijing 100021, China; 3 Nanjing Geneseeq Technology Inc., Nanjing 210032, China; 4 Cancer Hospital of Huanxing Chaoyang District Beijing, Beijing 100021, China

**Keywords:** Anlotinib, angiogenesis, HER2-negative, breast cancer, ctDNA

## Abstract

**Objective:**

Anlotinib is a novel tyrosine kinase inhibitor blocking angiogenesis. This study was performed to assess the efficacy and safety of anlotinib in patients with metastatic breast cancer.

**Methods:**

Patients with HER2-negative breast cancer, who were pre-treated with anthracycline or taxanes in a neoadjuvant, adjuvant, or metastatic setting, and had treatment failure after at least one prior chemotherapy regimen in the metastatic setting were enrolled. Anlotinib was administered at 12 mg daily for 14 days in a 21-day cycle until disease progression or unacceptable toxicity occurred. Simultaneously, 5–10 mL of venous blood was collected to perform circulating tumor DNA (ctDNA) testing every 2 treatment cycles. The primary endpoint was the objective response rate (ORR). Secondary endpoints included the disease control rate (DCR), progression-free survival (PFS), overall survival, safety, and biomarkers.

**Results:**

Twenty-six eligible patients were enrolled, with a median age of 56 (30–75) years. The median follow-up time was 10.5 months. The ORR was 15.4%, the DCR was 80.8%, and the median PFS was 5.22 months (95% confidence interval 2.86–6.24). Fourteen (53.8%) patients survived for more than 10 months. The changes in the detectable ctDNA variant allele frequency were consistent with the tumor response. The most common treatment-related adverse events were hypertension (57.7%), thyroid-stimulating hormone elevation (34.6%), and hand-foot syndrome (23.1%).

**Conclusion:**

Anlotinib showed objective efficacy with tolerable toxicity in heavily pre-treated, metastatic HER2-negative breast cancer. The dynamic changes in the ctDNA variant allele fraction may be predictive of the tumor response.

## Introduction

Among women worldwide, breast cancer is the malignant tumor with the highest incidence and the second leading cause of cancer-related death^1^. Approximately 20%–30% of patients with early breast cancer eventually develop metastatic breast cancer, and the median survival time for metastatic breast cancer ranges from 2 to 3 years^2–4^. In China, HER2-negative breast cancer accounts for approximately 65% of all breast cancers^5^. After adequate endocrine therapy and targeted therapy, the treatment options for hormone receptor-positive/HER2-negative breast cancer are similar to those of triple-negative breast cancer (TNBC). In contrast to HER2-positive breast cancer, for HER2-negative breast cancer, there are no specific targeted drugs or consensus recommendations regarding the choice of regimen after first-line treatment in the metastatic setting^6,7^. New drugs are urgently needed for the treatment of metastatic HER2-negative breast cancer, particularly for second or higher lines of treatment after metastasis.

Tumor angiogenesis plays an important role in tumor growth and invasion^8,9^. Bevacizumab is an anti-angiogenic monoclonal antibody that has shown some efficacy alone and in combination with chemotherapy for metastatic breast cancer^10–13^. Sorafenib, sunitinib, and apatinib are anti-angiogenic small-molecule tyrosine kinase inhibitors (TKIs) that mainly target vascular endothelial growth factor receptor (VEGFR)-1 (Flt1), VEGFR-2 (KDR), VEGFR-3 (Flt4), platelet-derived growth factor receptors (PDGFRs), and c-KIT. Numerous clinical studies have examined the application of anti-angiogenic drugs in breast cancer. However, sorafenib treatment alone has not been found to yield any improvement in progression-free survival (PFS)^14,15^, and sunitinib has limitations, owing to serious adverse events (AEs) in breast cancer^16^. In addition, apatinib has demonstrated potential efficacy in the treatment of metastatic breast cancer^17,18^. Notably, to date, none of these drugs have been recommended by any key international guidelines for breast cancer.

Similarly, anlotinib is a new type of anti-angiogenic small-molecule TKI whose major targets are VEGFR1-3, FGFR1-4, PDGFR-α, PDGFR-β, and stem cell factor receptors, which inhibit tumor angiogenesis and growth^19–21^. In clinical applications, anlotinib has exhibited excellent efficacy against various solid tumors in phase I and III studies, with manageable toxicity^19,22–24^. However, data on anlotinib in the treatment of metastatic breast cancer remain lacking. This study therefore aimed to explore the efficacy, safety, and related biomarkers of anlotinib in metastatic TNBC or hormone receptor-positive/HER2-negative breast cancer in patients who received chemotherapy and endocrine therapy, with targeted therapy available to them in the metastatic setting.

## Materials and methods

### Patients

This study was a single-center, single-arm phase II clinical study. We enrolled patients between 18 and 75 years of age with HER2-negative metastatic breast cancer, who were previously treated with anthracycline and taxane based chemotherapy (in the neoadjuvant, adjuvant, or metastatic setting) and at least one line of chemotherapy for TNBC in the metastatic setting, with at least one line of chemotherapy and all endocrine therapy available for hormone receptor-positive breast cancer after metastasis. Other inclusion criteria were an Eastern Cooperative Oncology Group (ECOG) score of 0–2, with measurable lesions defined according to Response Evaluation Criteria in Solid Tumors (RECIST) v1.1 criteria.

All enrolled patients signed informed consent forms. The trial was approved by the Ethics Committee of the Cancer Hospital, Chinese Academy of Medical Sciences, registered with ClinicalTrials.gov (NCT04002284), and conducted according to the principles of the Declaration of Helsinki.

### Study design and procedures

Patients were given 12 mg anlotinib once daily for 14 consecutive days in a 21-day cycle. Computed tomography or magnetic resonance imaging were used to evaluate treatment efficacy every 6 weeks until disease progression or unacceptable toxicity. Blood pressure was monitored twice daily during the first 3 weeks and at least once per day after the blood pressure stabilized. Routine blood tests were performed every week; physical examination, evaluation of liver and kidney function, and an electrocardiogram were performed every 3 weeks. Simultaneously, 5–10 mL blood was collected for ctDNA testing before the treatment and every 2 cycles until withdrawal of anlotinib. Adjustment of the dose and interruption of treatment because of AEs were permitted, although the duration of drug interruption could not exceed 14 days. AEs were graded with Common Terminology Criteria for Adverse Events (CTCAE) version 4.0.

### Outcomes

The primary endpoint was the objective response rate (ORR), and the secondary endpoints were the disease control rate (DCR), PFS, overall survival (OS), safety, and ctDNA biomarkers.

The ORR was defined as the proportion of patients who achieved complete response (CR) and partial response (PR). The DCR was defined as the proportion of patients who achieved CR, PR, or stable disease (SD). PFS was defined as the duration from the start of treatment to the last follow-up in patients with disease progression (PD) or to death from any cause, whichever occurred first. OS was defined as the duration from the beginning of treatment until death due to any cause.

### Circulating tumor DNA sequencing

Genomic DNA was extracted from patient plasma samples and subjected to library construction according to published protocols^25^. Hybridization capture-based targeted next-generation sequencing with a panel of 425 cancer-relevant genes was performed on the Illumina HiSeq platform (detected genes listed in **Supplementary Table S1**). Genomic alterations were analyzed as previously described^25^.

Detected SNVs and INDELs were further filtered with the following criteria: i) minimum ≥ 5 variant supporting reads and variant allele fraction (VAF) ≥ 1%, ii) filtered out if present in > 1% population frequency in the 1000 Genomes Project or ExAC database, iii) filtered out through an internal database of recurrent sequencing errors (≥ 3 variant reads and ≤ 20% VAF in at least 30 of ˜2,000 normal control samples) on the same sequencing platform. This assay was validated in compliance with the College of American Pathologists (CAP) and Clinical Laboratory Improvement Amendments (CLIA) with a limit of detection of 1% VAF.

### Statistical analysis

Simon’s minimax two-stage design was used with a one-sided α error of 0.05 and a power of 80%^26^. In preliminary experiments, we estimated that the ORR with anlotinib alone was 16%, in contrast to the 3% ORR of the placebo. Under these conditions, at least 17 patients would be included in the first stage, and the second stage would include 9 more patients. For a total sample size of 26 patients, if at least 3 patients had a response, this treatment would be considered a success.

Patients who received ≥ 1 cycle of anlotinib were included in survival and safety analyses. The data cut-off date for analyses was March 22, 2020. The Kaplan-Meier method was used to estimate PFS and OS. ORR/DCR comparison was performed with Fisher’s exact test. The 95% confidence interval (CI) of ORR/DCR was calculated with the Clopper-Pearson method. PFS was compared between 2 groups with a two-sided log-rank test. The hazard ratio (HR) and 95% CI were estimated with the Cox proportional-hazards model, with the receptor status or other clinicopathological factors as a single covariate. GraphPad Prism version 7.0 and SAS software version 9.4 were used for graphing and data analysis.

## Results

### Patient characteristics

From July 1, 2018, to January 10, 2020, 26 patients with metastatic TNBC or hormone receptor-positive/HER2-negative breast cancer were enrolled. The median follow-up time was 10.5 (2.5–18.5) months (data cut-off day of March 22, 2020). The median age was 56 (30–75) years. In total, 61.5% of the patients were hormone receptor positive/HER2-negative, and 10 (38.5%) patients were TNBC. All hormone receptor-positive/HER2-negative patients received at least first-line endocrine therapy and first-line chemotherapy after metastasis. Because of drug availability and economic considerations, 3 (of 16, 18.8%) patients received CDK4/6 inhibitor and mTOR inhibitor treatment. Two (of 16, 12.5%) patients received only CDK4/6 inhibitor or mTOR inhibitor treatment combined with endocrine drugs. The median number of previous systematic lines of treatment (including endocrine therapy and chemotherapy) in the metastatic setting was 2 (1–8). In total, 84.6% of patients had received fluorouracil treatment, 38.6% had received platinum treatment, and 65.4% had received other drug treatments, including vinorelbine, gemcitabine, and etoposide. The basic clinical characteristics are shown in **Table 1**.

**Table 1:** Patient characteristics at baseline

Characteristics	Anlotinib		
	Total (*n* = 26)	Hormone receptor positive (*n* = 16)	Hormone receptor negative (*n* = 10)
Age, median (range)	56 (30–75)	56 (30–75)	50 (32–64)
Age (years), *n* (%)			
≥ 65	3 (11.54)	3 (18.75)	0
< 65	23 (88.46)	13 (81.25)	10 (100)
ECOG, *n* (%)			
0	7 (26.92)	5 (31.25)	2 (20.00)
1	16 (61.54)	10 (62.50)	6 (60.00)
2	3 (11.54)	1 (6.25)	2 (20.00)
Hormone receptor, *n* (%)			
Positive	16 (61.54)	16 (100)	0
Negative	10 (38.46)	0	10 (100)
Type of metastatic site, *n* (%)			
Non-visceral	4 (15.38)	2 (12.50)	2 (20.00)
Visceral	22 (84.62)	14 (87.50)	8 (80.00)
Number of metastatic sites, *n* (%)			
1	3 (11.54)	2 (12.50)	1 (10.00)
2	12 (46.15)	7 (43.75)	5 (50.00)
≥ 3	11 (42.31)	7 (43.75)	4 (40.00)
Metastatic site, *n* (%)			
Lymph nodes	13 (50.00)	7 (43.75)	6 (60.00)
Liver	9 (34.62)	8 (50.00)	1 (10.00)
Lung	17 (65.38)	9 (56.25)	8 (80.00)
Pleural effusion	6 (23.08)	3 (18.75)	3 (30.00)
Chest wall	2 (7.69)	1 (6.25)	1 (10.00)
Pericardial effusion	2 (7.69)	0	2 (20.00)
Bone	15 (57.69)	12 (75.00)	3 (30.00)
Neoadjuvant, *n* (%)			
Yes	3 (11.54)	1 (6.25)	2 (20.00)
No	23 (88.46)	15 (93.75)	8 (80.00)
Adjuvant chemotherapy, *n* (%)			
Yes	23 (88.46)	15 (93.75)	10 (100)
No	3 (11.54)	1 (6.25)	0
Adjuvant endocrine therapy, *n* (%)			
Yes	16 (61.54)	16 (100)	0
No	10 (38.46)	0	10 (100)
Previous lines of systematic treatment, *n* (%)			
≤ 2	14 (53.85)	8 (50.00)	6 (60.00)
≥ 3	12 (46.15)	8 (50.00)	4 (40.00)
Type of previous endocrine therapy combined with target therapy, *n* (%)			
Both CDK/4/6 inhibitor and mTOR inhibitor	3 (11.54)	3 3 (18.75)	0
Only CDK4/6 inhibitor	2 (7.69)	2 (12.50)	0
Only mTOR inhibitor	2 (7.69)	2 (12.50)	0
Previous chemotherapy after metastasis, *n* (%)			
Taxanes	26 (100)	16 (100)	10 (100)
Fluorouracil^†^	22 (84.62)	12 (75.00)	10 (100)
Platinum	10 (38.46)	3 (18.75)	7 (70.00)
Others^‡^	17 (65.38)	10 (62.50)	7 (70.00)

ECOG, Eastern Cooperative Oncology Group.†Including capecitabine and S-1. ‡Other drugs, including gemcitabine, vinorelbine, and etoposide.

### Treatment efficacy

**Figure 1:**
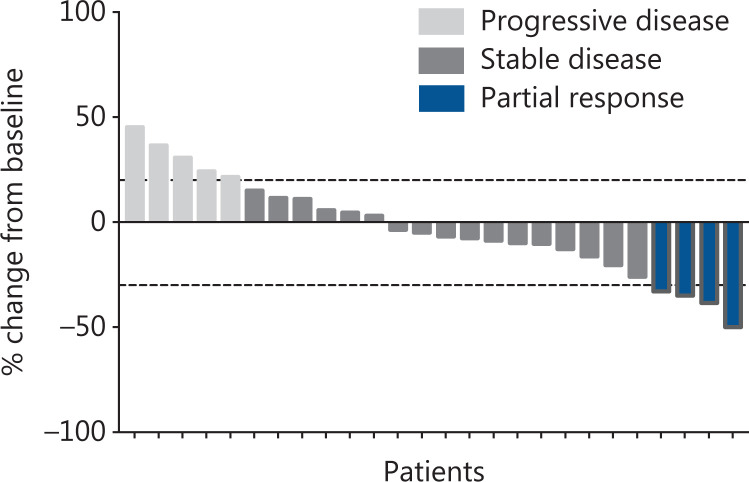
Waterfall plot of the best percentage change in target lesion size.

**Figure 2:**
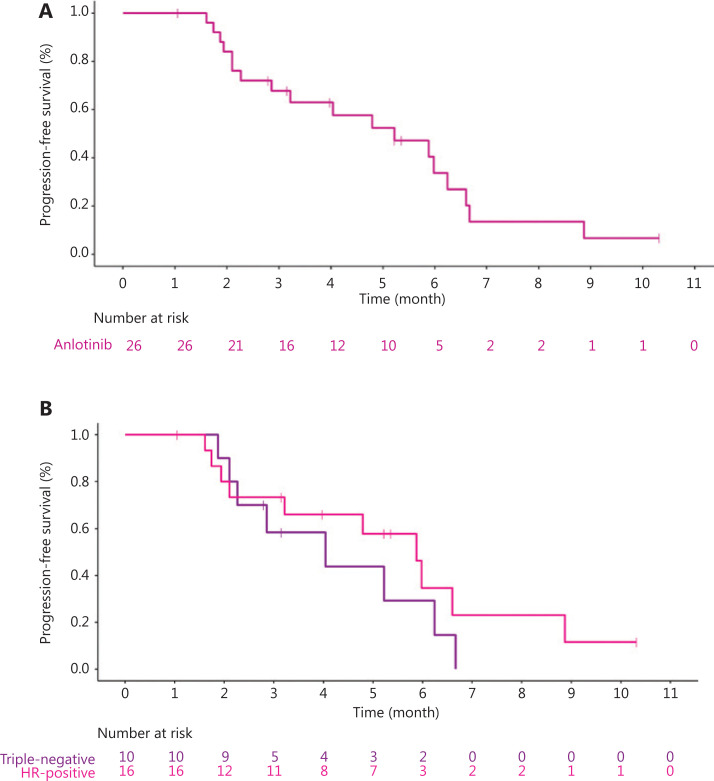
Kaplan-Meier graph showing progression-free survival. (A) The median progression-free survival (PFS) of all patients (*n* = 26) was 5.22 months. (B) The median PFS of hormone receptor-positive (*n* = 16) and hormone receptor-negative (*n* = 10) patients was 5.88 months and 4.04 months, respectively, HR = 0.62, 95% CI (0.24–1.63), *P* = 0.32.

Eighteen (69.2%) patients discontinued treatment because of disease progression, 4 (15.4%) patients died, and 4 (15.4%) patients were lost to follow-up. No treatment-related deaths were observed. Fifteen (57.7%) patients had varying degrees of tumor shrinkage (**Figure 1**). Four (15.4%) patients achieved PR, and the ORR was 15.4% (4/26). For hormone receptor-positive patients, the ORR was 18.8% (95% CI 4.05–45.65), and for TNBC patients, the ORR was 10.0% (95% CI 0.25–44.50). Seventeen (65.4%) patients had SD, and the DCR was 80.8% (21/26). For hormone receptor-positive patients, the DCR was 87.5% (95% CI 61.65–98.45). For TNBC patients, the DCR was 70.0% (95% CI 34.75–93.33). Comparisons between groups are shown in **Table 2**. The median PFS was 5.22 months (95% CI 2.86–6.24) (**Figure 2A**): 5.88 months (95% CI 1.94–8.87) for hormone receptor-positive patients and 4.04 months (95% CI 1.87–6.24) for TNBC (**Figure 2B**). The median OS has not yet been determined, but 14 (14/26, 53.8%) patients have survived for more than 10 months. There was no significant difference in the ORR, DCR, or PFS between the hormone receptor-positive and hormone receptor-negative groups. Subgroup analysis showed that factors such as hormone receptors, the number of lines of treatment, ECOG status, and the presence of visceral metastasis did not significantly affect the median PFS (**Supplementary Table S2**).

**Table 2:** Treatment response

	Total	Hormone receptor positive	Hormone receptor negative	Statistics
Numbers	26	16	10	
ORR (%)	15.38	18.75	10	
95% CI	(4.36–34.87)	(4.05–45.65)	(0.25–44.50)	*P* = 1.00^†^
DCR (%)	80.77	87.5	70	
95% CI	(60.65–93.45)	(61.65–98.45)	(34.75–93.33)	*P* = 0.34^‡^
Numbers censoring, *n* (%)	8 (30.77)	6 (37.50)	2 (20.00)	
Median PFS	5.22	5.88	4.04	
95% CI	(2.86–6.24)	(1.94–8.87)	(1.87–6.24)	
HR^§^	–	–	–	0.62, *χ*^2^ = 0.9595
95% CI^§^	–	–	–	(0.24–1.63), *P* = 0.32

PFS, progression-free survival; CI, confidence interval; HR, hazard ratio; ORR, objective response rate; DCR, disease control rate.†The ORR/DCR comparison between groups (positive *vs.* triple negative) was analyzed with Fisher’s exact test. ‡The 95% CI of the ORR/DCR was calculated with the Clopper-Pearson method. §The comparison of progression-free survival between 2 groups (hormone receptor positive *vs.* hormone receptor negative) was performed with a log-rank test. The HR and 95% CI (hormone receptor positive *vs.* hormone receptor negative) were estimated with the Cox proportional-hazards model.

**Table 3:** Summary of treatment-related adverse events

Adverse events	All grades,* n* (%)	Grade 1,* n* (%)	Grade 2,* n* (%)	Grades 3–4,* n* (%)
Fatigue	4 (15.38)	3 (11.54)	1 (3.85)	0 (0)
Anorexia	2 (7.69)	0 (0)	2 (7.69)	0 (0)
Weight loss	1 (3.85)	1 (3.85)	0 (0)	0 (0)
Pharyngalgia	3 (11.54)	3 (11.54)	0 (0)	0 (0)
Mucositis oral	1 (3.85)	1 (3.85)	0 (0)	0 (0)
Cough	2 (7.69)	2 (7.69)	0 (0)	0 (0)
Hand-foot syndrome	6 (23.08)	5 (19.23)	0 (0)	1 (3.85)
Urinary tract infection	1 (3.85)	0 (0)	1 (3.85)	0 (0)
Hematuria	2 (7.69)	2 (7.69)	0 (0)	0 (0)
Proteinuria	4 (15.38)	4 (15.38)	0 (0)	0 (0)
Hypertension	15 (57.69)	4 (15.38)	4 (15.38)	7 (26.92)
TSH elevation	9 (34.62)	9 (34.62)	0 (0)	0 (0)
Hypothyroidism	2 (7.69)	2 (7.69)	0 (0)	0 (0)
Hypertriglyceridemia	1 (3.85)	1 (3.85)	0 (0)	0 (0)
Hypercholesterolemia	1 (3.85)	1 (3.85)	0 (0)	0 (0)
LDL elevation	2 (7.69)	2 (7.69)	0 (0)	0 (0)
Alanine aminotransferase	2 (7.69)	1 (3.85)	1 (3.85)	0 (0)
Aspartate aminotransferase	1 (3.85)	0 (0)	1 (3.85)	0 (0)

LDL, low-density lipoprotein; TSH, thyroid-stimulating hormone.

### Safety

No treatment-related deaths were observed. Most AEs were mild to moderate (**Table 2**). All patients received anlotinib at 12 mg once per day for 14 days in a 21-day cycle. The most common AEs were hypertension (15/26, 57.8%), elevated thyroid-stimulating hormone (9/26, 34.6%), and hand-foot syndrome (6/26, 23.1%). Grade 3–4 AEs were hypertension (7/26, 26.9%) and hand-foot syndrome (1/26, 3.8%). No other serious AEs were observed. One (3.8%) patient discontinued treatment because of grade 3 hand-foot syndrome, and after the anlotinib was decreased to 10 mg/d, the symptoms of hand-foot syndrome returned to grade 1. Subgroup analysis showed that patients who had hand-foot syndrome had a longer median PFS than those who did not [median PFS 5.22 months (95% CI 2.27–6.60) *vs.* 2.86 months (95% CI 2.10–NE), HR 1.41 (95% CI 0.37–5.39), *P* = 0.62], but this finding was not statistically significant. The ORR, DCR, and PFS did not differ in patients with or without hypertension, proteinuria, hand-foot syndrome, or thyroid-stimulating hormone (TSH) elevation (**Supplementary Table S2**).

### Gene alterations in ctDNA

**Figure 3:**
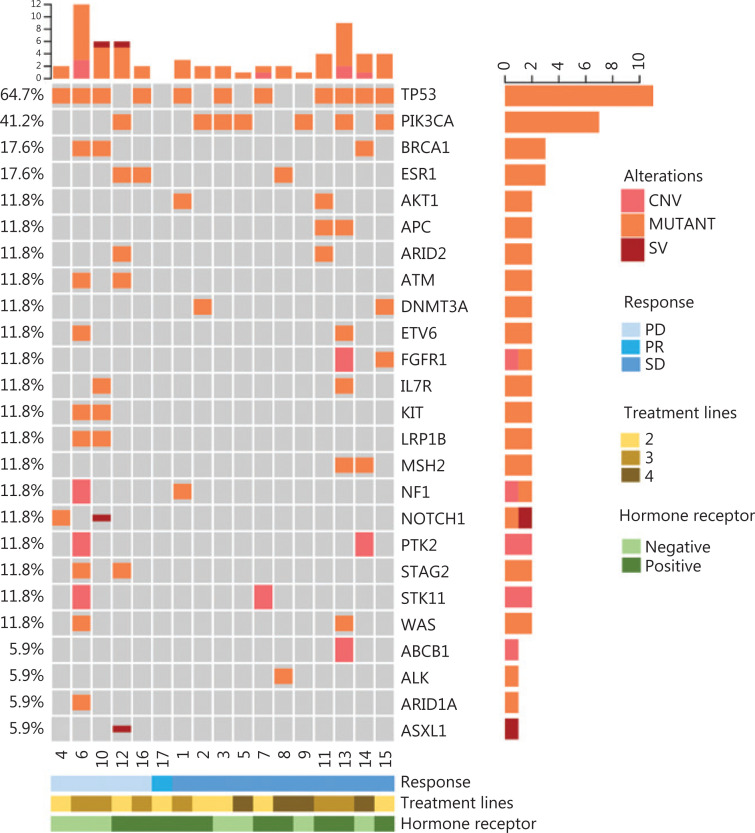
Distribution of the top 25 genomic alterations in the entire population at baseline.

During treatment, 17 patients had baseline blood collection, and 11 patients had serial blood collected for ctDNA analysis. Baseline ctDNA analysis showed that 16 patients had different numbers of ctDNA alterations and varying degrees of ctDNA VAF. The median number of alterations was 4 (1–24). The top 25 frequently mutated genes are shown in **Figure 3**. The types of gene alterations were mainly copy number variations, point mutations, and structural variations (SVs). The most commonly mutated genes were *TP53* (11/17, 64.7%), *PIK3CA* (7/17, 41.2%), *BRCA1* (3/16, 17.6%), and *ESR1* (3/16, 17.6%). Subgroup analysis showed that the median PFS of patients with detected SVs in the ctDNA was 1.74 months (95% CI 1.61–1.87), whereas that of patients with no SVs was 5.88 months (95% CI 2.10–6.67) (*P* = 0.0004) (**Supplementary Figure S1A**). The median PFS of the patients who had *TP53* mutations in their baseline ctDNA (2.27 months *vs.* 6.60 months, *P* = 0.057) or *PIK3CA* mutations with more than 1% VAF (1.74 months *vs.* 5.88 months, *P* = 0.0065) was significantly shorter than that of patients without these mutations (**Supplementary Figure S1B, C**). The differences in the ORR and DCR among patients with detected SVs, *TP53*, and *PIK3CA* are displayed in **Supplementary Table S2**. Among 11 patients with serial ctDNA detection, 6 had significantly higher ctDNA VAF at disease progression, and 2 had significantly lower ctDNA VAF when they showed tumor shrinkage with imaging, whereas 2 showed a slight increase in ctDNA VAF as the disease progressed (**Supplementary Figure S2A–S2J**). Notably, no ctDNA alterations were detected in one of the patients at baseline and after 2 cycles, and the computed tomography evaluation showed that she had PR. In total, 81.8% (9/11) of patients had changes in ctDNA VAF levels consistent with the efficacy evaluation, thereby suggesting that dynamic changes in ctDNA VAF levels may be a predictor of treatment efficacy.

## Discussion

This is the first study exploring the safety, efficacy, and biomarkers of anlotinib in heavily pre-treated metastatic HER2-negative breast cancer. Our research showed that anlotinib may have potential efficacy in patients with metastatic HER2-negative breast cancer receiving at least one regimen after metastasis.

Anlotinib is a multi-target tyrosinase inhibitor that blocks tumor angiogenesis by inhibiting VEGFR/FGFR/PDGFR and can also restrain tumor cell proliferation by blockade of FGFR/c-KIT^20,21,27^. Preclinical studies have shown that anlotinib induces hepatoma cell apoptosis by activating the Erk and Akt pathways^28^; targets the GINS1 gene and consequently regulates synovial sarcoma cell proliferation^29^; and blocks the MET pathway, thus inhibiting osteosarcoma angiogenesis^30^.

The potential anticancer effects and good safety of anlotinib have been demonstrated in a phase I clinical study conducted in our hospital^19^. Anlotinib has additionally shown good efficacy in phase II and III clinical trials in non-small cell lung cancer, sarcoma, and other tumours^22–24^. However, because anti-angiogenic drugs have low efficacy and high toxicity in breast cancer, their clinical application is limited. Studies have shown that use of bevacizumab alone for the treatment of metastatic breast cancer results in an ORR of 9.3%^31^. The small-molecule multi-target anti-angiogenic drug sorafenib results in an ORR of 2%, a DCR of 39%^14^, and a median PFS of 2 months in patients with metastatic breast cancer^15^. Additionally, in these patients, the ORR with sunitinib is 11%, and the median time to progression is 2.5 months^16^. Our study included patients who had received a median of 2 treatment lines with ECOG ≤ 2; the ORR was 15.4%, and DCR was 80.8%. The median PFS was 5.2 months, and the rate of patient survival for more than 10-months was 54%. Our research suggests that anlotinib has potential efficacy in treating metastatic HER2-negative breast cancer. The hormone receptor-positive patients enrolled in this study received at least one course of endocrine therapy, chemotherapy, and CDK4/6 plus mTOR inhibitor treatment, as long as the drugs were available and affordable. Subsequently, their treatment options were similar to those for patients with triple-negative advanced breast cancer; therefore, our study included HER2-negative metastatic breast cancer without restriction of hormone receptor status. In this study, the differences among the ORR, DCR, and median PFS between hormone receptor-positive and triple-negative patients were not statistically significant, but the median PFS of hormone receptor-positive patients was slightly longer than that of triple-negative patients (**Supplementary Table S2**). However, whether there is a difference in the efficacy of anlotinib between hormone receptor-positive and hormone receptor-negative patients must be confirmed by a larger study. Moreover, anlotinib is given orally, and therefore it is convenient to administer, results in decreased hospitalization time, and is cost effective.

No new AEs occurred in this study. All AEs were consistent with those reported for other tumors, such as non-small cell lung cancer and sarcoma^22,23^. Of note, no other serious AEs were observed in this study, except for hypertension and hand-foot syndrome, thus suggesting that anlotinib is safe in patients with metastatic breast cancer.

The pre-treatment ctDNA testing in this study showed that most patients had different degrees of alterations. The most commonly mutated genes were *TP53* and *PIK3CA*. We found that patients with *TP53* mutations and a *PIK3CA* VAF of more than 1% in the ctDNA had significantly shorter PFS (**Supplementary Figure S1B, S1C**), thus indicating that *TP53* and *PIK3CA* mutations may be poor prognostic factors for patients with breast cancer, as validated in several previous studies^32–35^. The types of alterations detected in this study were point mutations, copy number variations, and SVs. SVs are large fragment mutations on chromosomes, and mainly include the insertion and deletion of large chromosome fragments, the inversion of a certain area within a chromosome, and inter-chromosome translocation between 2 chromosomes^36^. We found that the occurrence rate of SVs was 7.8%, and patients with SVs detected by ctDNA screening had a lower PFS (**Supplementary Figure S1A**), thus suggesting that the existing gene SVs may be poor prognostic factors for breast cancer.

As a real-time liquid biopsy technique, ctDNA screening has been found to be a reliable method for predicting therapeutic efficacy^37^. In our study, the dynamic changes in ctDNA levels were comparable to the changes in imaging findings in 81% of patients (**Supplementary Figure S2**), thereby suggesting that ctDNA monitoring might be used as a potential tool for prediction of treatment efficacy; however, this conclusion must be confirmed by a large-sample prospective randomized controlled study.

## Conclusions

In summary, anlotinib is effective and well tolerated in heavily pre-treated HER2-negative metastatic breast cancer. Changes in ctDNA VAF levels may be predictive of the efficacy of anlotinib. Anlotinib, an anti-angiogenic TKI, normalizes tumor angiogenesis, alters tumor blood perfusion, and has potential for use in combined chemotherapy or targeted therapy for metastatic breast cancer.

## References

[1] Siegel RL, Miller KD, Jemal A (2019). Cancer statistics, 2019. CA Cancer J Clin.

[2] Mayer EL, Burstein HJ (2007). Chemotherapy for metastatic breast cancer. Hematol Oncol Clin North Am.

[3] Stuckey A (2011). Breast cancer: epidemiology and risk factors. Clin Obstet Gynecol.

[4] Mariotto AB, Etzioni R, Hurlbert M, Penberthy L, Mayer M (2017). Estimation of the number of women living with metastatic breast cancer in the United States. Cancer Epidemiol Biomarkers Prev.

[5] Wang Q, Li J, Zheng S, Li JY, Pang Y, Huang R (2012). Breast cancer stage at diagnosis and area-based socioeconomic status: a multicenter 10-year retrospective clinical epidemiological study in China. BMC Cancer.

[6] Cardoso F, Senkus E, Costa A, Papadopoulos E, Aapro M, Andre F (2018). 4th ESO-ESMO international consensus guidelines for advanced breast cancer (ABC 4) dagger. Ann Oncol.

[7] Network NCC (2019). NCCN clinical practice guidelines in Oncology (NCCN Guidelines®) Breast Cancer Version 3.

[8] Folkman J (1971). Tumor angiogenesis: therapeutic implications. N Engl J Med.

[9] Banerjee S, Dowsett M, Ashworth A, Martin LA (2007). Mechanisms of disease: angiogenesis and the management of breast cancer. Nat Clin Pract Oncol.

[10] Miles D, Cameron D, Bondarenko I, Manzyuk L, Alcedo JC, Lopez RI (2017). Bevacizumab plus paclitaxel *vs.* placebo plus paclitaxel as first-line therapy for HER2-negative metastatic breast cancer (MERiDiAN): a double-blind placebo-controlled randomised phase III trial with prospective biomarker evaluation. Eur J Cancer.

[11] Miller K, Wang M, Gralow J, Dickler M, Cobleigh M, Perez EA (2007). Paclitaxel plus bevacizumab vs. paclitaxel alone for metastatic breast cancer. N Engl J Med.

[12] Robert NJ, Dieras V, Glaspy J, Brufsky AM, Bondarenko I, Lipatov ON (2011). RIBBON-1: randomized, double-blind, placebo-controlled, phase III trial of chemotherapy with or without bevacizumab for first-line treatment of human epidermal growth factor receptor 2-negative, locally recurrent or metastatic breast cancer. J Clin Oncol.

[13] Brufsky AM, Hurvitz S, Perez E, Swamy R, Valero V, O’Neill V (2011). RIBBON-2: a randomized, double-blind, placebo-controlled, phase III trial evaluating the efficacy and safety of bevacizumab in combination with chemotherapy for second-line treatment of human epidermal growth factor receptor 2-negative metastatic breast cancer. J Clin Oncol.

[14] Bianchi G, Loibl S, Zamagni C, Salvagni S, Raab G, Siena S (2009). Phase II multicenter, uncontrolled trial of sorafenib in patients with metastatic breast cancer. Anticancer Drugs.

[15] Moreno-Aspitia A, Morton RF, Hillman DW, Lingle WL, Rowland KM, Wiesenfeld M (2009). Phase II trial of sorafenib in patients with metastatic breast cancer previously exposed to anthracyclines or taxanes: North Central Cancer Treatment Group and Mayo Clinic Trial N0336. J Clin Oncol.

[16] Burstein HJ, Elias AD, Rugo HS, Cobleigh MA, Wolff AC, Eisenberg PD (2008). Phase II study of sunitinib malate, an oral multitargeted tyrosine kinase inhibitor, in patients with metastatic breast cancer previously treated with an anthracycline and a taxane. J Clin Oncol.

[17] Hu X, Zhang J, Xu B, Jiang Z, Ragaz J, Tong Z (2014). Multicenter phase II study of apatinib, a novel VEGFR inhibitor in heavily pretreated patients with metastatic triple-negative breast cancer. Int J Cancer.

[18] Hu X, Cao J, Hu W, Wu C, Pan Y, Cai L (2014). Multicenter phase II study of apatinib in non-triple-negative metastatic breast cancer. BMC Cancer.

[19] Sun Y, Niu W, Du F, Du C, Li S, Wang J (2016). Safety, pharmacokinetics, and antitumor properties of anlotinib, an oral multi-target tyrosine kinase inhibitor, in patients with advanced refractory solid tumors. J Hematol Oncol.

[20] Lin B, Song X, Yang D, Bai D, Yao Y, Lu N (2018). Anlotinib inhibits angiogenesis via suppressing the activation of VEGFR2, PDGFRbeta and FGFR1. Gene.

[21] Taurin S, Yang CH, Reyes M, Cho S, Coombs DM, Jarboe EA (2018). Endometrial cancers harboring mutated fibroblast growth factor receptor 2 protein are successfully treated with a new small tyrosine kinase inhibitor in an orthotopic mouse model. Int J Gynecol Cancer.

[22] Han B, Li K, Wang Q, Zhang L, Shi J, Wang Z (2018). Effect of anlotinib as a third-line or further treatment on overall survival of patients with advanced non-small cell lung cancer: The ALTER 0303 phase 3 randomized clinical trial. JAMA Oncol.

[23] Chi Y, Fang Z, Hong X, Yao Y, Sun P, Wang G (2018). Safety and efficacy of anlotinib, a multikinase angiogenesis inhibitor, in patients with refractory metastatic soft-tissue sarcoma. Clin Cancer Res.

[24] Sun Y, Du F, Gao M, Ji Q, Li Z, Zhang Y (2018). Anlotinib for the treatment of patients with locally advanced or metastatic medullary thyroid cancer. Thyroid.

[25] Yang Z, Yang N, Ou Q, Xiang Y, Jiang T, Wu X (2018). Investigating novel resistance mechanisms to third-generation EGFR tyrosine kinase inhibitor osimertinib in non-small cell lung cancer patients. Clin Cancer Res.

[26] Simon R (1989). Optimal two-stage designs for phase II clinical trials. Control Clin Trials.

[27] Xie C, Wan X, Quan H, Zheng M, Fu L, Li Y (2018). Preclinical characterization of anlotinib, a highly potent and selective vascular endothelial growth factor receptor-2 inhibitor. Cancer Sci.

[28] He C, Wu T, Hao Y (2018). Anlotinib induces hepatocellular carcinoma apoptosis and inhibits proliferation via Erk and Akt pathway. Biochem Biophys Res Commun.

[29] Tang L, Yu W, Wang Y, Li H, Shen Z (2019). Anlotinib inhibits synovial sarcoma by targeting GINS1: a novel downstream target oncogene in progression of synovial sarcoma. Clin Transl Oncol.

[30] Wang G, Sun M, Jiang Y, Zhang T, Sun W, Wang H (2019). Anlotinib, a novel small molecular tyrosine kinase inhibitor, suppresses growth and metastasis via dual blockade of VEGFR2 and MET in osteosarcoma. Int J Cancer.

[31] Cobleigh MA, Langmuir VK, Sledge GW, Miller KD, Haney L, Novotny WF (2003). A phase I/II dose-escalation trial of bevacizumab in previously treated metastatic breast cancer. Semin Oncol.

[32] Kim JY, Park K, Jung HH, Lee E, Cho EY, Lee KH (2016). Association between mutation and expression of TP53 as a potential prognostic marker of triple-negative breast cancer. Cancer Res Treat.

[33] Meric-Bernstam F, Zheng X, Shariati M, Damodaran S, Wathoo C, Brusco L (2018). Survival outcomes by TP53 mutation status in metastatic breast cancer. JCO Precis Oncol.

[34] Mosele F, Stefanovska B, Lusque A, Tran Dien A, Garberis I, Droin N (2020). Outcome and molecular landscape of patients with PIK3CA-mutated metastatic breast cancer. Ann Oncol.

[35] Cristofanilli M, DeMichele A, Giorgetti C, Turner NC, Slamon DJ, Im SA (2018). Predictors of prolonged benefit from palbociclib plus fulvestrant in women with endocrine-resistant hormone receptor-positive/human epidermal growth factor receptor 2-negative metastatic breast cancer in PALOMA-3. Eur J Cancer.

[36] Gawronski AR, Lin YY, McConeghy B, LeBihan S, Asghari H, Kockan C (2019). Structural variation and fusion detection using targeted sequencing data from circulating cell free DNA. Nucleic Acids Res.

[37] Rossi G, Mu Z, Rademaker AW, Austin LK, Strickland KS, Costa RLB (2018). Cell-free DNA and circulating tumor cells: comprehensive liquid biopsy analysis in advanced breast cancer. Clin Cancer Res.

