# Supplementary material for: Anlotinib has good efficacy and low toxicity: a phase II study of anlotinib in pre-treated HER-2 negative metastatic breast cancer
Source: Cancer Biol Med. 2021 Aug 15;18(3):849–59. doi: 10.20892/j.issn.2095-3941.2020.0463 (PMC8330540; doi:10.20892/j.issn.2095-3941.2020.0463)
Supplement: Supplementary file 1 [file cbm-18-849-s001.pdf]

Supplementary materials

Table S1 Genes identified in the panel of 425 cancer-relevant genes

Number	Gene name	Number	Gene name	Number	Gene name
1	*ABCB1* (*MDR1*)	143	*FANCM*	285	*PDK1*
2	*ABCB4*	144	*FAT1*	286	*PGR*
3	*ABCC2* (*MRP2*)	145	*FBXW7*	287	*PHOX2B*
4	*ADH1A*	146	*FGF19*	288	*PIK3C3*
5	*ADH1B*	147	*FGFR1*	289	*PIK3CA*
6	*ADH1C*	148	*FGFR2*	290	*PIK3R1*
7	*AIP*	149	*FGFR3*	291	*PIK3R2*
8	*AKT1*	150	*FGFR4*	292	*PKHD1*
9	*AKT2*	151	*FH*	293	*PLAG1*
10	*AKT3*	152	*FLCN*	294	*PLK1*
11	*ALDH2*	153	*FLT1* (*VEGFR1*)	295	*PMS1*
12	*ALK*	154	*FLT3*	296	*PMS2*
13	*AMER1*	155	*FLT4*	297	*POLD1*
14	*APC*	156	*FOXA1*	298	*POLD3*
15	*AR*	157	*FOXP1*	299	*POLE*
16	*ARAF*	158	*FRG1*	300	*POLH*
17	*ARID1A*	159	*GATA1*	301	*POT1*
18	*ARID1B*	160	*GATA2*	302	*PPARD*
19	*ARID2*	161	*GATA3*	303	*PPP2R1A*
20	*ARID5B*	162	*GATA4*	304	*PRDM1*
21	*ASCL4*	163	*GATA6*	305	*PRF1*
22	*ASXL1*	164	*GNA11*	306	*PRKACA*
23	*ATF1*	165	*GNAQ*	307	*PRKACG*
24	*ATIC*	166	*GNAS*	308	*PRKAR1A*
25	*ATM*	167	*GRIN2A*	309	*PRKCI*
26	*ATR*	168	*GRM3*	310	*PRKDC*
27	*ATRX*	169	*GRM8*	311	*PRSS1*
28	*AURKA*	170	*GSTM1*	312	*PRSS3*
29	*AURKB*	171	*GSTM4*	313	*PTCH1*
30	*AXIN2*	172	*GSTM5*	314	*PTEN*
31	*AXL*	173	*GSTP1*	315	*PTK2*
32	*B2M*	174	*GSTT1*	316	*PTPN11*

Table S1 Continued

Number	Gene name	Number	Gene name	Number	Gene name
33	*BAD*	175	*HDAC2*	317	*PTPN13*
34	*BAI3*	176	*HDAC9*	318	*PTPRD*
35	*BAK1*	177	*HGF*	319	*QKI*
36	*BAP1*	178	*HLA-A*	320	*RAC1*
37	*BARD1*	179	*HNF1A*	321	*RAC3*
38	*BAX*	180	*HNF1B*	322	*RAD50*
39	*BCL2*	181	*HRAS*	323	*RAD51*
40	*BCL2L11* (*BIM*)	182	*HSD3B1*	324	*RAD51B*
41	*BCR*	183	*IDH1*	325	*RAD51C*
42	*BIRC3*	184	*IDH2*	326	*RAD51D*
43	*BLM*	185	*IFNG*	327	*RAD54L*
44	*BMPR1A*	186	*IFNGR1*	328	*RAF1*
45	*BRAF*	187	*IGF1R*	329	*RARA*
46	*BRCA1*	188	*IGF2*	330	*RARG*
47	*BRCA2*	189	*IKBKE*	331	*RASGEF1A*
48	*BRD4*	190	*IKZF1*	332	*RB1*
49	*BRIP1*	191	*IL7R*	333	*RECQL4*
50	*BTG2*	192	*INPP4B*	334	*RELN*
51	*BTK*	193	*IRF2*	335	*RET*
52	*BUB1B*	194	*JAK1*	336	*RHOA*
53	*c11orf30*	195	*JAK2*	337	*RICTOR*
54	*CASP8*	196	*JAK3*	338	*RNF43*
55	*CBL*	197	*JARID2*	339	*ROS1*
56	*CBLB*	198	*JUN*	340	*RPTOR*
57	*CCND1*	199	*KDM5A*	341	*RRM1*
58	*CCNE1*	200	*KDM6A*	342	*RUNX1*
59	*CD274* (*PD-L1*)	201	*KDR* (*VEGFR2*)	343	*RUNX1T1*
60	*CD74*	202	*KEAP1*	344	*SBDS*
61	*CDA*	203	*KIF1B*	345	*SDC4*
62	*CDC73*	204	*KIF5B*	346	*SDHA*
63	*CDH1*	205	*KIT*	347	*SDHB*
64	*CDK10*	206	*KITLG*	348	*SDHC*
65	*CDK12*	207	*KLLN*	349	*SDHD*
66	*CDK4*	208	*KMT2A* (*MLL*)	350	*SEPT9*

Table S1 Continued

Number	Gene name	Number	Gene name	Number	Gene name
67	*CDK6*	209	*KMT2B*	351	*SETBP1*
68	*CDK8*	210	*KMT2C*	352	*SETD2*
69	*CDKN1A*	211	*KMT2D*	353	*SF3B1*
70	*CDKN1B*	212	*KRAS*	354	*SGK1*
71	*CDKN1C*	213	*LHCGR*	355	*SLC34A2*
72	*CDKN2A*	214	*LMO1*	356	*SLC3A2*
73	*CDKN2B*	215	*LRP1B*	357	*SLC7A8*
74	*CDKN2C*	216	*LYN*	358	*SMAD2*
75	*CEBPA*	217	*LZTR1*	359	*SMAD3*
76	*CEP57*	218	*MAP2K1* (*MEK1*)	360	*SMAD4*
77	*CHD4*	219	*MAP2K2* (*MEK2*)	361	*SMAD7*
78	*CHEK1*	220	*MAP2K4*	362	*SMARCA4*
79	*CHEK2*	221	*MAP3K1*	363	*SMARCB1*
80	*CREBBP*	222	*MAP3K4*	364	*SMO*
81	*CRKL*	223	*MAP4K3*	365	*SOS1*
82	*CSF1R*	224	*MAX*	366	*SOX1*
83	*CTCF*	225	*MCL1*	367	*SOX14*
84	*CTLA4*	226	*MDM2*	368	*SOX2*
85	*CTNNB1*	227	*MDM4*	369	*SOX21*
86	*CUL3*	228	*MECOM*	370	*SPOP*
87	*CUX1*	229	*MED12*	371	*SPRY4*
88	*CXCR4*	230	*MEF2B*	372	*SRC*
89	*CYLD*	231	*MEN1*	373	*SRY*
90	*CYP19A1*	232	*MET*	374	*STAG2*
91	*CYP2A13*	233	*MGMT*	375	*STAT3*
92	*CYP2A6*	234	*MITF*	376	*STK11*
93	*CYP2A7*	235	*MLH1*	377	*STMN1*
94	*CYP2B6*6*	236	*MLH3*	378	*STT3A*
95	*CYP2C19*2*	237	*MLLT1*	379	*SUFU*
96	*CYP2C9*3*	238	*MLLT3*	380	*TAP1*
97	*CYP2D6*	239	*MLLT4*	381	*TAP2*
98	*CYP3A4*4*	240	*MPL*	382	*TEK*
99	*CYP3A5*	241	*MRE11A*	383	*TEKT4*
100	*DAXX*	242	*MSH2*	384	*TERC*

Table S1 Continued

Number	Gene name	Number	Gene name	Number	Gene name
101	*DDR2*	243	*MSH6*	385	*TERT*
102	*DENND1A*	244	*MTHFR*	386	*TET2*
103	*DHFR*	245	*MTOR*	387	*TGFBR2*
104	*DICER1*	246	*MUTYH*	388	*THADA*
105	*DLL3*	247	*MYC*	389	*TMEM127*
106	*DNMT3A*	248	*MYCL*	390	*TMPRSS2*
107	*DPYD*	249	*MYCN*	391	*TNFAIP3*
108	*DUSP2*	250	*MYD88*	392	*TNFRSF11A*
109	*EGFR*	251	*MYH9*	393	*TNFRSF14*
110	*EML4*	252	*NAT1*	394	*TNFRSF19*
111	*EP300*	253	*NBN*	395	*TNFSF11*
112	*EPAS1*	254	*NCOR1*	396	*TOP1*
113	*EPCAM*	255	*NF1*	397	*TOP2A*
114	*EPHA2*	256	*NF2*	398	*TP53*
115	*EPHA3*	257	*NFE2L2*	399	*TP63*
116	*EPHA5*	258	*NFKBIA*	400	*TPMT*
117	*EPHB2*	259	*NKX2-1*	401	*TSC1*
118	*ERBB2* (*HER2*)	260	*NKX2-4*	402	*TSC2*
119	*ERBB2IP*	261	*NOTCH1*	403	*TSHR*
120	*ERBB3*	262	*NOTCH2*	404	*TTF1*
121	*ERBB4*	263	*NOTCH3*	405	*TUBB3*
122	*ERCC1*	264	*NPM1*	406	*TUBB4A*
123	*ERCC2*	265	*NQO1*	407	*TUBB4B*
124	*ERCC3*	266	*NRAS*	408	*TUBB6*
125	*ERCC4*	267	*NRG1*	409	*TYMS*
126	*ERCC5*	268	*NSD1*	410	*U2AF1*
127	*ESR1*	269	*NTRK1*	411	*UGT1A1*
128	*ETV1*	270	*NTRK2*	412	*VAMP2*
129	*ETV4*	271	*NTRK3*	413	*VEGFA*
130	*ETV6*	272	*PAK3*	414	*VHL*
131	*EWSR1*	273	*PALB2*	415	*WAS*
132	*EXT1*	274	*PALLD*	416	*WISP3*
133	*EXT2*	275	*PARK2*	417	*WRN*
134	*EZH2*	276	*PARP1*	418	*WT1*

Table S1 Continued

Number	Gene name	Number	Gene name	Number	Gene name
135	*FANCA*	277	*PARP2*	419	*XPA*
136	*FANCC*	278	*PAX5*	420	*XPC*
137	*FANCD2*	279	*PBRM1*	421	*XRCC1*
138	*FANCE*	280	*PDCD1* (*PD1*)	422	*YAP1*
139	*FANCF*	281	*PDCD1LG2* (*PD-L2*)	423	*ZNF2*
140	*FANCG*	282	*PDE11A*	424	*ZNF217*
141	*FANCI*	283	*PDGFRA*	425	*ZNF703*
142	*FANCL*	284	*PDGFRB*		

Table S2 Univariate analysis of ORR, DCR, and PFS

Characteristics		*n*	ORR†				DCR†				PFS†			
			Response (CR/PR) *n* (%)	Odds ratio‡ (95% CI)	*P*	FDR§	Control (SD/CR/PR) *n* (%)	Odds ratio‡ (95% CI)	*P*	FDR§	Median (95% CI)	HR (95% CI)	*P*	FDR§
Age (years)	≥ 65	3	1 (33.33)	3.33 (0.23,49.09)	0.4077	1	2 (66.67)	0.42 (0.03,5.85)	0.4885	0.8402	1.74 (1.61,5.98)	0.32 (0.09,1.17)	0.0855	0.6141
	< 65	23	3 (13.04)				19 (82.61)				5.22 (2.86,6.60)			
ECOG	1–2	19	1 (5.26)	0.07 (0.01,0.91)	0.0468	0.8422	14 (73.68)	0.18 (0.01,3.62)	0.2782	0.8402	4.04 (2.10,5.98)	0.38 (0.12,1.20)	0.0977	0.6141
	0	7	3 (42.86)				7 (100)				6.60 (1.74,NE)			
Hormone receptor	Negative	10	1 (10.00)	0.48 (0.04,5.40)	1	1	7 (70.00)	0.33 (0.05,2.48)	0.3402	0.8402	4.04 (1.87,6.24)	0.62 (0.24,1.63)	0.3331	0.8582
	Positive	16	3 (18.75)				14 (87.50)				5.88 (1.94,8.87)			
Type of metastatic site	Non-visceral	4	0 (0)	0.46 (0.02,10.12)	1	1	3 (75.00)	0.67 (0.05,8.19)	1	1	5.73 (2.10,8.87)	1.40 (0.44,4.50)	0.5731	0.8597
	Visceral	22	4 (18.18)				18 (81.82)				5.22 (2.27,6.24)			
Number of metastases	2	12	1 (8.33)	0.05 (0.00,1.07)	0.0813	0.8422	10 (83.33)	0.60 (0.02,15.76)	1	1	4.80 (1.74,8.87)	0.60 (0.07,5.02)	0.6341	0.8597
	1	3	2 (66.67)				3 (100)				5.98 (NE,NE)			
	≥ 3	11	1 (9.09)	0.05 (0.00,1.18)	0.0934	0.8422	8 (72.73)	0.35 (0.01,8.63)	1	1	4.22 (1.87,6.24)	0.31 (0.04,2.49)	0.2693	0.8464
	1	3	2 (66.67)				3 (100)				5.98 (NE,NE)			
	≥ 3	11	1 (9.09)	1.10 (0.06,20.01)	1	1	8 (72.73)	0.58 (0.09,3.71)	0.6404	0.8883	4.22 (1.87,6.24)	0.52 (0.18,1.53)	0.2335	0.7903
	2	12	1 (8.33)				10 (83.33)				4.80 (1.74,8.87)			
Metastatic site														
Chest wall	No	24	4 (16.67)	1.10 (0.05,27.01)	1	1	19 (79.17)	0.71 (0.03,17.06)	1	1	4.80 (2.27,6.24)	0.70 (0.16,3.15)	0.6448	0.8597
	Yes	2	0 (0)				2 (100)				6.28 (5.88,6.67)			
Liver	No	17	4 (23.53)	6.33 (0.30,132.05)	0.2631	0.8979	14 (82.35)	1.33 (0.18,9.91)	1	1	5.88 (2.86,6.67)	2.04 (0.73,5.68)	0.1719	0.7624
	Yes	9	0 (0)				7 (77.78)				2.66 (1.74,6.60)			
Bone	No	11	2 (18.18)	1.44 (0.17,12.23)	1	1	11 (100)	12.05 (0.59,245.16)	0.0527	0.6744	5.98 (2.86,6.67)	1.16 (0.44,3.06)	0.7713	0.8894
	Yes	15	2 (13.33)				10 (66.67)				4.01 (1.94,6.60)			
Lung	No	9	0 (0)	0.16 (0.01,3.29)	0.2631	0.8979	7 (77.78)	0.75 (0.10,5.58)	1	1	4.80 (1.74,6.67)	0.94 (0.36,2.46)	0.8993	0.9229
	Yes	17	4 (23.53)				14 (82.35)				5.22 (2.86,6.24)			
Lymph nodes	No	13	4 (30.77)	12.79 (0.61,266.54)	0.0957	0.8422	12 (92.31)	5.33 (0.51,56.24)	0.3217	0.8402	5.98 (1.74,NE)	1.51 (0.58,3.92)	0.399	0.8582
	Yes	13	0 (0)				9 (69.23)				4.80 (2.10,6.24)			

Table S2 Continued

Characteristics		*n*	ORR†				DCR†				PFS†			
			Response (CR/PR) *n* (%)	Odds ratio‡ (95% CI)	*P*	FDR§	Control (SD/CR/PR) *n* (%)	Odds ratio‡ (95% CI)	*P*	FDR§	Median (95% CI)	HR (95% CI)	*P*	FDR§
Pleural effusion	No	20	3 (15.00)	0.88 (0.07,10.46)	1	1	15 (75.00)	0.22 (0.01,4.52)	0.2981	0.8402	5.88 (2.10,6.67)	1.52 (0.51,4.49)	0.4515	0.8597
	Yes	6	1 (16.67)				6 (100)				4.63 (1.74,6.24)			
Pericardial effusion	No	24	4 (16.67)	1.10 (0.05,27.01)	1	1	19 (79.17)	0.71 (0.03,17.06)	1	1	4.80 (2.27,6.24)	0.70 (0.16,3.15)	0.6448	0.8597
	Yes	2	0 (0)				2 (100)				6.28 (5.88,6.67)			
Neoadjuvant chemotherapy	No	23	4 (17.39)	1.62 (0.07,37.15)	1	1	19 (82.61)	2.38 (0.17,33.00)	0.4885	0.8402	5.22 (2.27,6.24)	1.52 (0.43,5.40)	0.519	0.8597
	Yes	3	0 (0)				2 (66.67)				2.86 (2.10,6.60)			
Adjuvant chemotherapy	No	3	0 (0)	0.62 (0.03,14.24)	1	1	2 (66.67)	0.42 (0.03,5.85)	0.4885	0.8402	5.22 (2.10,8.87)	1.13 (0.32,4.06)	0.8485	0.9229
	Yes	23	4 (17.39)				19 (82.61)				4.80 (2.27,6.24)			
Previous lines of systematic treatment	≥ 3	12	3 (25.00)	4.33 (0.39,48.60)	0.3061	0.8979	11 (91.67)	4.40 (0.42,46.24)	0.3304	0.8402	5.98 (2.10,NE)	2.02 (0.75,5.41)	0.1627	0.7624
	≤ 2	14	1 (7.14)				10 (71.43)				3.22 (1.87,6.24)			
Adverse events														
Hypertension	No	11	1 (9.09)	0.40 (0.04,4.47)	0.6137	1	10 (90.91)	3.64 (0.35,38.22)	0.3562	0.8402	5.22 (1.61,6.60)	0.87 (0.34,2.22)	0.7751	0.8894
	Yes	15	3 (20.00)				11 (73.33)				4.80 (2.10,6.67)			
Proteinuria	No	22	4 (18.18)	2.19 (0.10,48.50)	1	1	17 (77.27)	0.35 (0.02,7.65)	0.5552	0.8842	5.88 (2.27,6.67)	1.40 (0.45,4.35)	0.5662	0.8597
	Yes	4	0 (0)				4 (100)				5.01 (2.10,6.60)			
Hand-foot syndrome	No	20	3 (15.00)	0.88 (0.07,10.46)	1	1	16 (80.00)	0.80 (0.07,8.91)	1	1	5.22 (2.27,6.60)	1.41 (0.37,5.39)	0.6173	0.8597
	Yes	6	1 (16.67)				5 (83.33)				2.86 (2.10,NE)			
TSH elevation	No	17	2 (11.76)	0.47 (0.05,4.03)	0.5906	1	13 (76.47)	0.41 (0.04,4.31)	0.6279	0.8883	5.22 (2.10,5.98)	0.94 (0.36,2.49)	0.9019	0.9229
	Yes	9	2 (22.22)				8 (88.89)				6.24 (1.94,6.67)			

Table S2 Continued

Characteristics		n	ORR[†]				DCR[†]				PFS[†]			
			Response (CR/PR) n (%)	Odds ratio[‡] (95% CI)	P	FDR[§]	Control (SD/CR/PR) n (%)	Odds ratio[‡] (95% CI)	P	FDR[§]	Median (95% CI)	HR (95% CI)	P	FDR[§]
ctDNA														
*TP53*_1 (VAF of ctDNA)	≥ 1%	8	0 (0)	0.33 (0.01,9.40)	1	1	5 (62.50)	0.52 (0.07,3.72)	0.6199	0.8883	2.27 (1.74,6.67)	0.62 (0.21,1.87)	0.395	0.8582
	< 1%	9	1 (11.11)				7 (77.78)				5.88 (1.61,6.60)			
	NE	9	3 (33.33)	3.05 (0.35,26.68)	0.5765	1	9 (100)	6.33 (0.26,152.84)	0.4706	0.8402	5.22 (2.10,5.98)	0.84 (0.24,2.92)	0.7859	0.8894
	< 1%	9	1 (11.11)				7 (77.78)				5.88 (1.61,6.60)			
	NE	9	3 (33.33)	9.15 (0.40,210.27)	0.2059	0.8979	9 (100)	12.09 (0.52,280.36)	0.0824	0.6744	5.22 (2.10,5.98)	1.36 (0.39,4.73)	0.628	0.8597
	≥ 1%	8	0 (0)				5 (62.50)				2.27 (1.74,6.67)			
*TP53*_2 (VAF of ctDNA)	> 0	10	0 (0)	0.21 (0.01,5.86)	0.4118	1	6 (60.00)	0.33 (0.04,2.84)	0.3382	0.8402	2.27 (1.74,6.24)	0.28 (0.07,1.04)	0.0567	0.499
	0	7	1 (14.29)				6 (85.71)				6.60 (1.61,NE)			
	NE	9	3 (33.33)	2.33 (0.26,21.06)	0.5846	1	9 (100)	4.38 (0.15,125.27)	0.4375	0.8402	5.22 (2.10,5.98)	0.44 (0.10,2.04)	0.2959	0.8582
	0	7	1 (14.29)				6 (85.71)				6.60 (1.61,NE)			
	NE	9	3 (33.33)	11.31 (0.50,256.17)	0.0867	0.8422	9 (100)	13.15 (0.60,288.28)	0.0867	0.6744	5.22 (2.10,5.98)	1.61 (0.51,5.07)	0.4164	0.8582
	> 0	10	0 (0)				6 (60.00)				2.27 (1.74,6.24)			
*PIK3CA*_1 (VAF of ctDNA)	≥ 1%	4	0 (0)	0.93 (0.03,27.12)	1	1	3 (75.00)	1.11 (0.12,10.15)	1	1	1.74 (1.61,4.04)	0.13 (0.03,0.61)	0.009	0.132
	< 1%	13	1 (7.69)				9 (69.23)				5.88 (2.10,6.67)			
	NE	9	3 (33.33)	4.49 (0.53,37.94)	0.2643	0.8979	9 (100)	9.00 (0.42,191.35)	0.115	0.7064	5.22 (2.10,5.98)	0.70 (0.21,2.39)	0.572	0.8597
	< 1%	13	1 (7.69)				9 (69.23)				5.88 (2.10,6.67)			
	NE	9	3 (33.33)	4.85 (0.20,118.60)	0.4965	1	9 (100)	8.14 (0.26,250.69)	0.3077	0.8402	5.22 (2.10,5.98)	5.26 (1.18,23.40)	0.0293	0.3223
	≥ 1%	4	0 (0)				3 (75.00)				1.74 (1.61,4.04)			
*PIK3CA*_2 (VAF of ctDNA)	> 0	6	0 (0)	0.54 (0.02,15.30)	1	1	5 (83.33)	2.20 (0.26,18.90)	0.6	0.8883	4.04 (1.61,6.24)	0.43 (0.12,1.53)	0.1906	0.7624
	0	11	1 (9.09)				7 (63.64)				5.88 (1.94,6.67)			
	NE	9	3 (33.33)	3.77 (0.44,32.31)	0.2848	0.8979	9 (100)	11.40 (0.53,246.67)	0.0941	0.6744	5.22 (2.10,5.98)	0.79 (0.23,2.71)	0.7134	0.8894
	0	11	1 (9.09)				7 (63.64)				5.88 (1.94,6.67)			
	NE	9	3 (33.33)	7.00 (0.30,164.39)	0.2286	0.8979	9 (100)	5.18 (0.18,150.43)	0.4	0.8402	5.22 (2.10,5.98)	1.86 (0.49,7.11)	0.3659	0.8582
	> 0	6	0 (0)				5 (83.33)				4.04 (1.61,6.24)			

Table S2 Continued

Characteristics		n	ORR[†]				DCR[†]				PFS[†]			
			Response (CR/PR) n (%)	Odds ratio[‡] (95% CI)	P	FDR[§]	Control (SD/CR/PR) n (%)	Odds ratio[‡] (95% CI)	P	FDR[§]	Median (95% CI)	HR (95% CI)	P	FDR[§]
Structural variation	Yes	2	0 (0)	1.93 (0.06,62.17)	1	1	0 (0)	0.06 (0.00,1.46)	0.0735	0.6744	1.74 (1.61,1.87)	0.03 (0.00,0.30)	0.0035	0.132
	No	15	1 (6.67)				12 (80.00)				5.88 (2.10,6.67)			
	NE	9	3 (33.33)	5.21 (0.62,43.57)	0.1304	0.8979	9 (100)	5.32 (0.24,115.85)	0.2663	0.8402	5.22 (2.10,5.98)	0.80 (0.25,2.59)	0.7095	0.8894
	No	15	1 (6.67)				12 (80.00)				5.88 (2.10,6.67)			
	NE	9	3 (33.33)	2.69 (0.10,73.20)	1	1	9 (100)	94.99 (1.48,NE)	0.0182	0.6744	5.22 (2.10,5.98)	32.20 (2.62,395.92)	0.0067	0.132
	Yes	2	0 (0)				0 (0)				1.74 (1.61,1.87)			

ORR, objective response rate; DCR, disease control rate; PFS, progression free survival; NE, not evaluated; PR, partial response; SD, stable disease; CR, complete response; CI, confidence interval; HR, hazard ratio; ECOG, Eastern Cooperative Oncology Group; TSH, thyroid stimulating hormone; FDR, false discovery rate.

†PFS was estimated with the Kaplan-Meier method. The HR and 95% CI were estimated with the Cox proportional-hazards model. ORR/DCR was compared with Fisher's exact test.

‡When calculating the odds ratio, if the value for a certain cell was 0, then 0.5 was added to each cell for adjustment.

§FDR: false discovery rate.

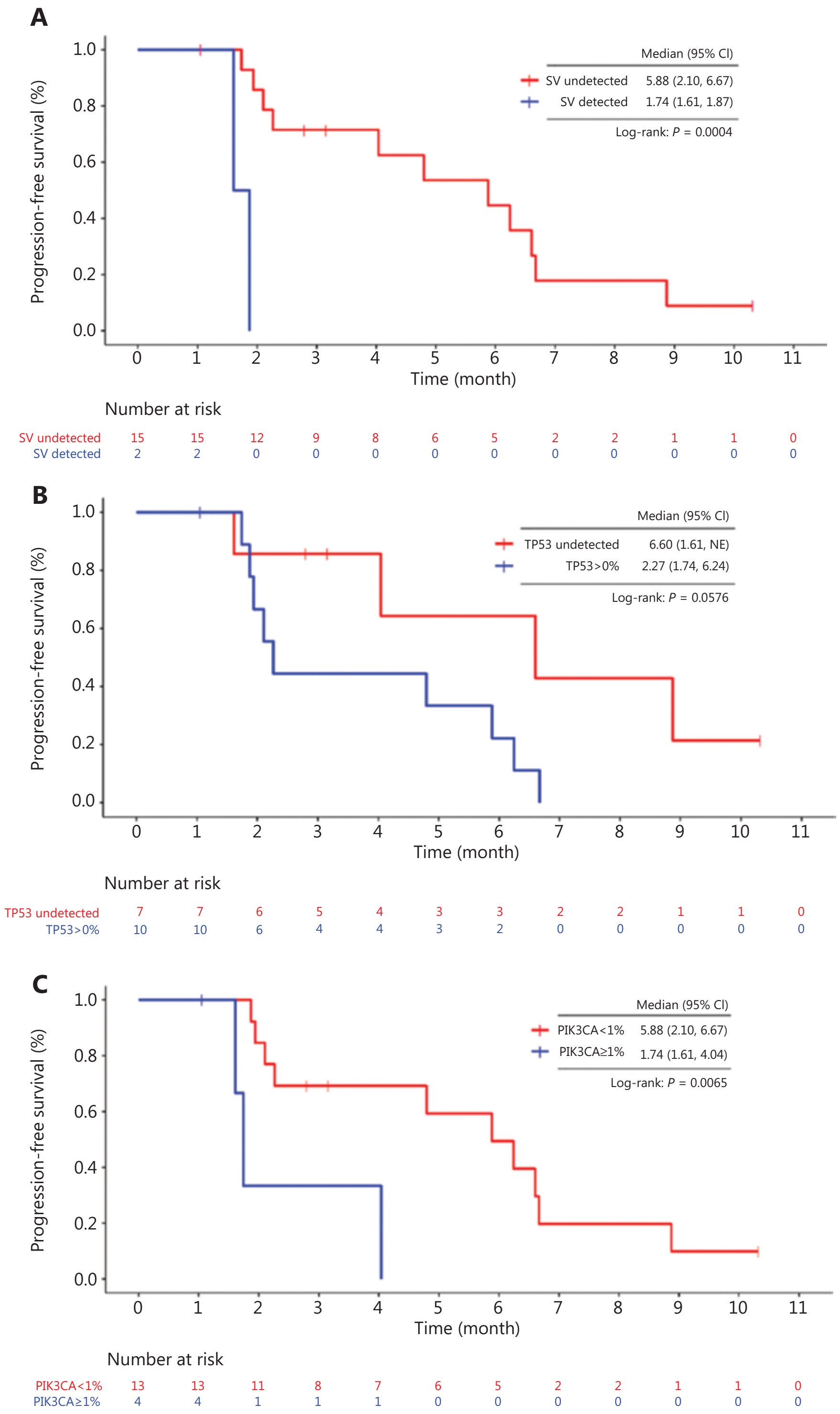

Figure S1 Kaplan-Meier curve of the PFS associated with different ctDNA alteration types or ctDNA levels at baseline. PFS, progression-free survival; ctDNA, circulating tumor DNA; HR, hazard ratio; CI, confidence interval; SV, structural variation; VAF, variant allelic frequency. The median PFS was significantly shorter for patients who had an SV as the detected alteration type in the ctDNA (1.74 *vs.* 5.88 months, *P* = 0.0004) (A), TP53 mutations (2.27 *vs.* 6.60 months, *P* = 0.0567) (B), and a ctDNA PIK3CA VAF of more than 1% (1.74 *vs.* 5.88 months, *P* = 0.0065) (C).

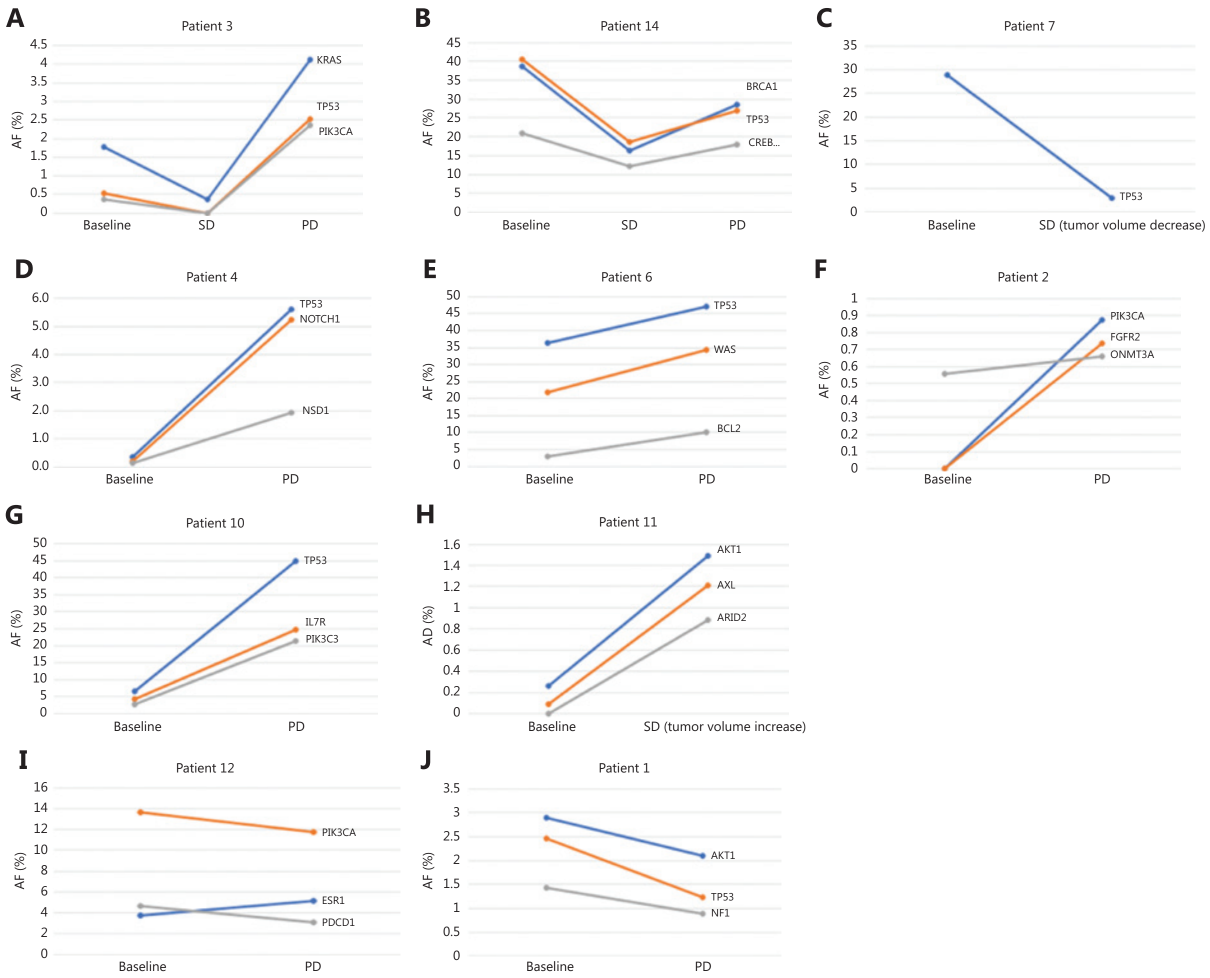

Figure S2 Serial ctDNA status of 10 patients. SD, stable disease; PD, progressive disease; VAF, variant allelic frequency. The ctDNA VAF changes in the top 3 genes in each patient are shown. The ctDNA VAF levels significantly decreased when the tumor loads were decreased, as shown in the A–C graphs (*n* = 3), whereas the ctDNA VAF levels increased when the tumor burden increased, as shown in the A–H graphs (*n* = 7). The ctDNA VAF levels slightly decreased when the tumor loads were increased, as shown in the I–J graphs (*n* = 2).
